# Fe_3_O_4_@Chitosan@ZIF-8@RVG29, an anti-glioma nanoplatform guided by fixed and activated by alternating magnetic field

**DOI:** 10.1038/s41598-024-57565-2

**Published:** 2024-03-24

**Authors:** Mohammad-Nabil Savari

**Affiliations:** Independent Researcher, Tehran, Iran

**Keywords:** Nanomedicine, Nanoscience and technology, Nanotoxicology, Cell-particle interactions, Medical toxicology, Methods of toxicology studies, Cancer therapy, Targeted therapies, Targeted therapies, CNS cancer, Drug development

## Abstract

There is considerable interest in developing anti-glioma nanoplatforms. They make the all-in-one combination of therapies possible. Here we show how the selective Glioblastoma multiforme (GBM) cell killing of the here-established nanoplatforms increased after each coating and how the here-established vibration-inducing Alternating magnetic field (AMF) decreased the treatment time from 72 h to 30 s. Thanks to their magnetite core, these nanoplatforms can be guided to the tumor's specific site by a Fixed magnetic field, they bypass the Blood–Brain Barrier (BBB) and accumulate at the tumor site thanks to the RVG29 bonding to the G-protein on the ion-gated channel receptor known as the nicotinic acetylcholine receptor (nAchR), which expresses on BBB cells and overexpresses on GBM cells, and thanks to the positive charge gained by both chitosan and RVG29's peptide. Both ZIF-8 and its mediate adherence, Chitosan increases the drug loading capacity that stimuli response to the tumor's acidic environment. The Zn^2+^ ions generated from ZIF-8 sustained degradation in such an environment kill the GBM cells. Dynamic Light Scattering (DLS) evaluated these nanoplatform's mean size 155 nm indicating their almost optimum size for brain applications. Based on their elements' intrinsic properties, these nanoplatforms can enhance and combine other adjuvant therapies.

## Introduction

The most prevalent and fatal brain tumor, glioblastoma multiforme (GBM), is still one of the most difficult and intimidating unresolved clinical issues in medicine^1^.

Conventional cancer treatments, like radiotherapy^2,3^, chemotherapy^2,3^, targeted therapy^2,3^, and surgery, have serious side effects and limited efficacy. In this case, we'll need a synergy between multiple monotherapies; which causes a super-additive effect ("1 + 1 > 2")^2–5^. It is more potent than any one monotherapy or their theoretical combination^5^. Combinated treatments, which use many synergistic approaches to treat cancer, have garnered a lot of interest lately^2,3^. For instance, multimodal synergistic therapies, which combine chemotherapy with other forms of treatment such as magnetic hyperthermia (MHT), photothermal therapy (PTT), and Photodynamic therapy (PDT), have the potential to minimize adverse effects, remove cancer entirely, and reduce the risk of recurrence while improving overall cancer treatment^2,3^.

Glioma cells express the ion-gated channel receptor known as the nicotinic acetylcholine receptor (nAchR)^6^. According to a study, glioma cells have 2.04 times more receptors on their surface than nonmalignant cells do^6^. This difference could encourage the accumulation of RVG29-modified Nano Particles (NPs) at the targeted region^6^. Additionally, nAchR expression is markedly elevated in hypoxic and ischemic environments, suggesting that nAchRs are also linked to angiogenesis^7^; alterations that are in line with the tumor microenvironment^2,4^. Tumor-specific delivery may be possible by targeting nAchR. The glycoprotein on the surface of the rabies virus, known as rabies virus glycoprotein (RVG), binds to the G-protein on the nAchR to infect mammalian neural cells^8^. pH affects nAchR-RVG binding; which, exhibits the highest affinity when pH is between 5.7 and 6.6^9^. A 29-amino acid sequence found in RVG competes with the snake venom toxin α-bungarotoxin (BTX) in interacting with acetylcholine receptors^9^. It was demonstrated that the RVG29 peptide bound to Neuro 2a cells that expressed nAchR but not to HeLa cells that did not express nAchR^10^. Studies have demonstrated that nAchR expression is significantly elevated in neuron cells in both pre-and post-synaptic sites as well as in the brain endothelial cells present in the blood–brain barrier (BBB), even though many drugs are prevented from passing through the BBB by the tight junctions formed by these cells^11,12^. RVG29 has an efficient rate of transporting genes, nucleic acids, or medications across BBB and through nAchR in brain endothelial cells^13,14^. Kwon et al., for instance, employed siRNA given via RVG29 to lessen the long-term effects of traumatic brain injury^14^. As a result, the RVG29 peptides can be used as a ligand for a delivery system that targets the brain.

Zeolite imidazolate framework-8 (ZIF-8) has low cytotoxicity, optimal drug delivery capability, acceptable drug loading, and biodegradability, making them promising drug delivery carriers^2,15,16^. ZIF-8 nanoparticles' sensitivity to pH^2,3,17^ allows them to be broken down and regulate the release of drugs in the acidic environments found in tumor cells and the surrounding tissue. Additionally, ZIF-8-based drug delivery systems^2,16^ offer combinatory tumor therapeutic approaches like photodynamic therapy^2,3^ and chemotherapy ^2,3,18^, chemotherapy and microwave thermal therapy^2,3,19^, chemo-photothermal therapy^2,3,20^, photodynamic- photothermal-, immuno-, and chemo-therapy^2,3,21^. In a study, M Xu et al. demonstrated that the pro-death effect caused by ZIF-8 was influenced by the release of zinc ions and the production of reactive oxygen species (ROS) as a result of ZIF-8 corrosion in the acidic compartments^22^. Fascinatingly, autophagy suppression effectively removed the production of ROS and free zinc ions, indicating that autophagic processes were involved in ZIF-8 corrosion at least in part. Furthermore, because Zn^2+^ is a positive regulator of both adaptive and innate immunity^23^, Zn^2+^ produced during ZIF-8 NPs breakdown in GBM cancer cells is thought to be an additional beneficial component for enhancing the immunological microenvironment that has been inhibited. As a result, the immune response to the loaded chemotherapy medicines that cause GBM cell pyroptosis will be strengthened.

(CS) Chitosan's backbone contains numerous functional groups, it can easily reform, and due to its sustained/controlled release properties, it can be conjugated to other polymeric NPs or loaded with medicines and imaging agents^2,24,25^. Chitosan may be taken into consideration as an extra therapeutic approach to prevent neuronal death in conditions linked to oxidative stress. Chitosan therefore has the potential to be employed for both the prevention and treatment of neurological illnesses^26^. Previous studies have described the use of chitosan particles as anti-glioma agent nanocarriers^27,28^.

Chitosan and magnetic nanoparticles (MNPs) can be coupled to enhance the MNPs' surface charge and enhance their uptake into tumors^2,25,29^. Compared to neutral-coated nanocomposites, mouse mesenchymal stem cells (MSCs) absorbed the 40 kDa-coated iron oxide-based nanocomposites six times more when the charge was increased from − 1.5 to + 18.2 mV^30^. Additionally, by counteracting the negative charges on a tumor cell's surface, positively charged chitosan nanoparticles (NPs) can aggregate in the tumor location, increasing medication concentration and enhancing anticancer action^31^. Furthermore, compared to monoZIF-8 coated particles, hybrid Chitosan-ZIF-8 coated nanoparticles are expected to exert stronger membrane contacts^32^. Magnetite (Fe_3_O_4_) with a large anisotropy constant (1.1 × 10^5^ ergs/cm^3^) and strong saturation magnetization has garnered significant attention^2,33^. A growing number of biomedical fields, such as magnetic resonance imaging (MRI)^2,24,25,33^, biological catalysis, magnetic hyperthermia^2,3,33^, magnetic targeting^2,3,25^, magnetic separation^2,3^, response-based therapy^2,3,33^, photography^2,3,33^, and drug delivery^2,16,25,33^, are using these as representative candidates of multifunctional nanomaterials^2,3^. Fe_3_O_4_ nanoparticles exhibit excellent performance as MRI contrast agents, but their drug-loading capacity is limited because the drug can only be bound on their surface. Additionally, using IONPs without a polymer coating and in unloaded conditions will cause precipitation or aggregation due to strong magnetic dipole–dipole attractions between particles, which means that their clinical application is restricted^2,29,34^. Consequently, different coatings^2,35^ have been proposed to increase the drug-loading capacity of Fe_3_O_4_ nanoparticles^2,16,25^. The subject of stimuli-responsive polymer coatings has seen many initiatives in recent years^2,3,16,33,35^. Changes in environmental stimuli, such as light, ionic strength, magnetic field, electric field, pH, and enzymes, cause these polymers to undergo a reversible phase transition and change their swelling behavior^2,3,17^. pH and temperature are two of these stimuli that have been employed extensively^2,3^. Typically, magnetic particles consist of a polymeric shell that offers advantageous functional groups and properties for a range of applications and a magnetic core that ensures a strong magnetic response^2,3,16,25^. To further increase the loading capacity and introduce some additional features such as responsiveness to stimuli and biodegradability, in this study, Fe_3_O_4_ nanoparticles were coated with ZIF-8 mediated with chitosan and were functionalized with RVG29 to be developed into a multifunctional nanoplatform against Glioblastoma multiforme.

## Results and discussion

### Fe_3_O_4_@CS FTIR characterization

**Figure 1:**
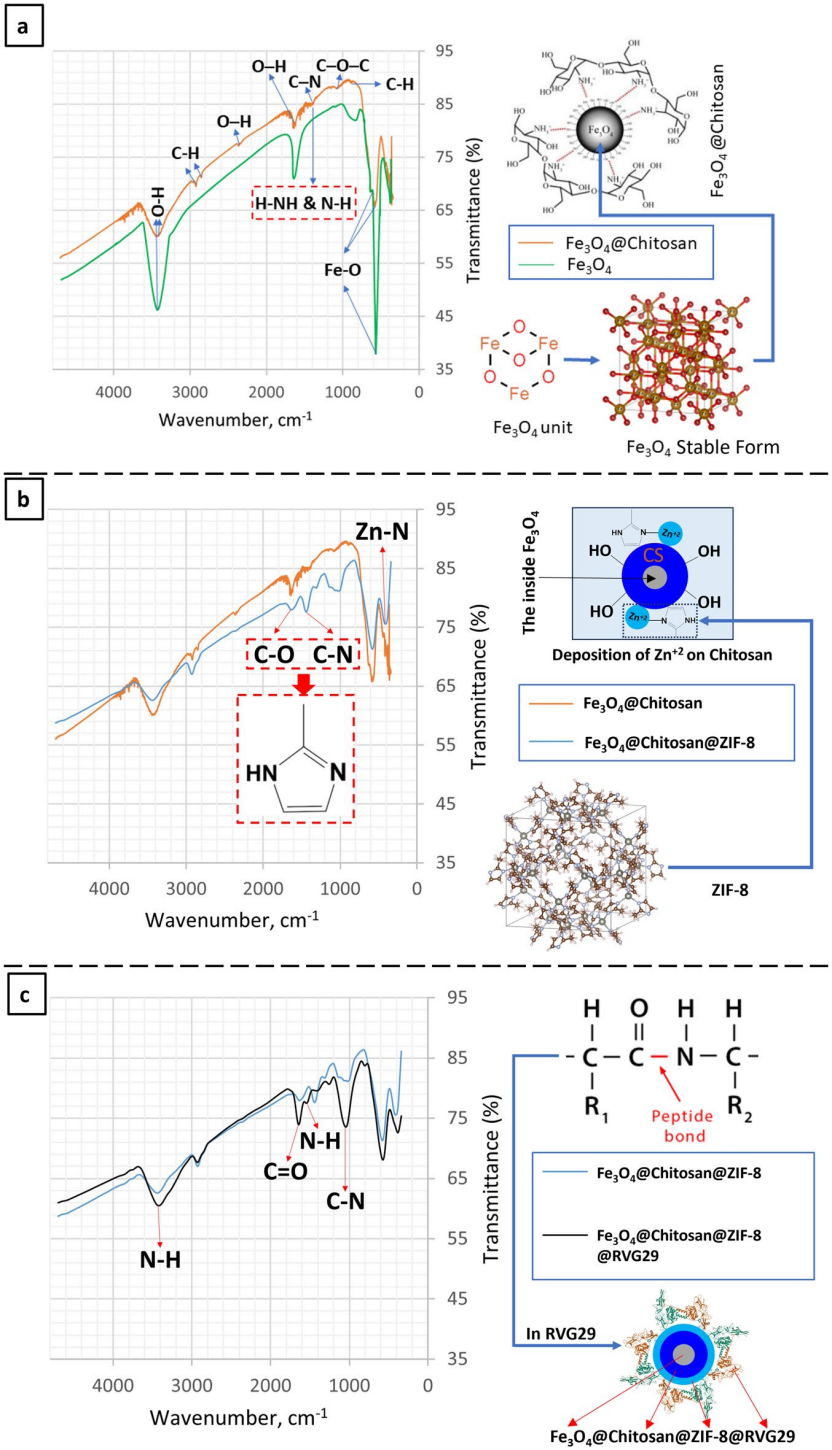
At left FTIR graph chart of the synthesized nanocomposites and the functional group of each wavelength peak, at right their related structure.

Figure 1a shows the Fourier transform infrared (FTIR) of bare Fe_3_O_4_ nanoparticles and the core–shell structure of Fe_3_O_4_@chitosan nanocomposites on the left and their simulated structure on the right. Each stable Fe_3_O_4_ cubic slab is made of 8 formula units^36^. The creation of magnetic nanoparticles is proved by the existence of three prominent absorption bands in all materials' FTIR at approximately 636, 581, and 370 cm^−1^. Additionally, the bands at 581 and 370 cm^−1^ were attributed to the Fe–O stretching vibration of the spinel structure's tetrahedral and octahedral cubic ferrite sites, respectively. Furthermore, the bands at 3405, 2345, and 1632 cm^−1^ are attributed to surface OH vibrations of the synthetic magnetic compounds. The molecules of ethylene glycol left behind the OH groups^37,38^ and, in comparison to Fe_3_O_4_@Chitosan, have greater vibrational intensities in Fe_3_O_4_ NPs. The lower vibrational strengths of these bands in Fe_3_O_4_@Chitosan suggest that the carbon composited had successfully protected the OH groups during glucose carbonization ^39^. The peaks at 2847 and 2936 cm^−1^ are ascribed to the symmetric and antisymmetric stretching vibration of C–H bonds that ethylenediamine molecules introduce during the solvothermal stage of the production of Fe_3_O_4_ nanoparticles^40,41^. The out-of-plane H–NH and N–H vibrational modes in conjunction with the antisymmetric C–N stretching vibrations were responsible for the weak to medium intensity peak seen at 1385 cm^−1^^42^. The in-plane and out-of-plane vibrational modes for residual C–H bond deformation were attributed to the peak at 870 cm^−1^^43^. A few of the peaks vanished, moved to different wavenumbers, or remained in the final nanocomposites. Remarkable alterations seen in the OH vibrations and Fe–O bonds at the tetrahedral cubic ferrite sites, to 580 cm^−1^ and 3437–3450 cm^−1^, respectively, suggest that the two nanocomposites maintained the spectral features of the original Fe_3_O_4_ nanoparticles. The strong connection between the encapsulating carbon layer bordering the surface of the original Fe_3_O_4_ nanoparticle and its core is indicated by the loss and shifting of some of the peaks in the nanocomposites' spectra. A desire for accumulation may be indicated by the absorption peak at 3442.09 cm^−1^, which could be caused by a significant number of hydroxyl groups of OH stretching vibrations. Less motivation to accumulate was evident in the core–shell structure as seen by the peak's lower intensity compared to the bare structure. FTIR spectra so unequivocally demonstrated that hydrogen bonding and van der Waals forces were the sources of chitosan coating.

### Fe_3_O_4_@CS@ZIF-8 FTIR characterization

ZIF-8's spectra (Fig. 1b showed distinctive peaks in the 1000–1800 cm^−1^ region, which corresponded to the imidazole ring's vibration^44^. Furthermore, the stretching vibrations of C–N were identified as the cause of the peak at 1471 cm^−1^^45^. Zn–N was represented by another distinctive peak at 409.89 cm^−1^^45^. The characteristic peaks of Fe_3_O_4_@Chitosan and ZIF-8 were both observed in the FTIR spectrum of Fe_3_O_4_@Chitosan@ZIF-8, indicating that ZIF-8 was successfully loaded on Chitosan.

### Fe_3_o_4_@Cs@Zif-8@Rvg29 FTIR characterization

Using infrared spectroscopy, the functionalization of the nanocomposite with the RVG29 peptide was verified. The bands depicted in Fig. 1c are indicative of the peptide bonds between amino acids found in the RVG29 peptide spectra. These bands correspond to the N–H stretching vibrations (1643.41 and 3421.83 cm^−1^)^46^, C = O stretching vibrations (1643.81 cm^−1^)^46^, and C-N stretching vibrations (573.84 and 1044.49 cm^–1^). The characteristic peaks of RVG29 and Fe_3_O_4_@Chitosan@ZIF-8 were both observed in the FTIR spectrum of Fe_3_O_4_@Chitosan@ZIF-8@RVG29, indicating that Fe_3_O_4_@Chitosan@ZIF-8 was successfully functionalized with RVG29.

### Particles’ structure

**Figure 2:**
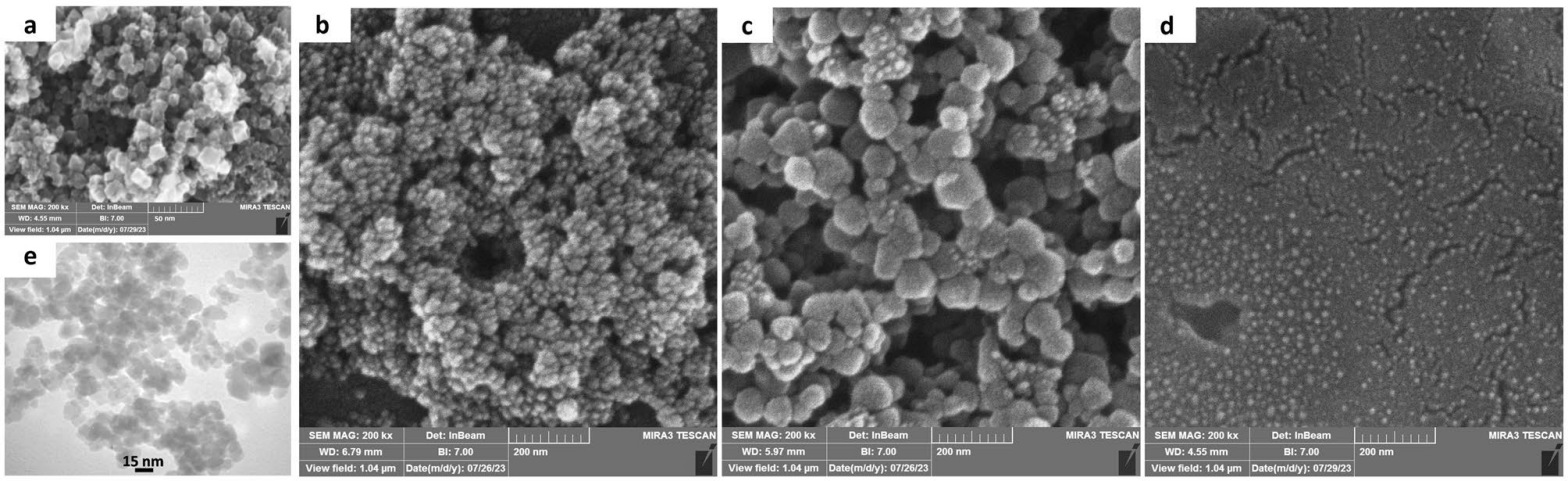
FE-SEM image of (**a**) Fe_3_O_4_ (**b**)Fe_3_O_4_@chitosan nanocomposites (**c**) Fe_3_O_4_@chitosan@ZIF-8 d) Fe_3_O_4_@Chitosan@ZIF-8@RVG29, And (**e**) TEM image of Fe_3_O_4_ synthesized in this study.

Parts a, b, c, and d of Fig. 2 show the FE-SEM Images of Fe_3_O_4_ at the size of 15 nm, Fe_3_O_4_@Chitosan at the size of 36 nm, Fe_3_O_4_@Chitosan@ZIF-8 at the size of 61 nm and Fe_3_O_4_@Chitosan@ZIF-8@RVG29 Nano platforms at the size of 150 nm, respectively. FE-SEM images indicate that the mentioned Nanocomposites were synthesized with homogenous sizes with almost spherical shapes. Part e shows a TEM Image of Fe_3_O_4_ indicating their unattached cubic crystal structure synthesis.

### Particles’ size

Every nanoparticle exhibited a mean size of less than 155 nm, making them appropriate for use in the brain and enabling the BBB bypass ^47,48^. Since Dynamic Light Scattering (DLS) calculates the hydrodynamic diameter of nanoparticles by taking into consideration the hydration sphere made up of water, ions, and counter-ions, it is conceivable that the size of the nanoparticles determined by DLS is slightly larger than that obtained by Electron Microscope (EM).

Coating the nanocomposite with each agent increases its size, as seen in Table 1, Fe_3_O_4_ had the lowest mean size and ranged from 10 to 25 nm, Fe_3_O_4_@Chitosan had the second lowest mean size and ranged from 21 to 75 nm, Fe_3_O_4_@Chitosan@ZIF-8 had the second biggest mean size and ranged from 32 to 99 nm and Fe_3_O_4_@Chitosan@ZIF-8@RVG29 had the biggest mean size and ranged from 120 to 240 nm. Concerning the polydispersity index, all values seem to not exceed 0.3, even after 4 months of storage, suggesting an acceptable size distribution^2,29^ with low variability and no aggregation of particles, typical of the high shear homogenization and ultrasonication methods^46^. Fe_3_O_4_@Chitosan@ZIF-8@RVG29 showed a 0.2 polydispersity index (lower than other nanoparticles) indicating that RVG29 functionalization enhanced the nanocomposite's polydispersity^2,29^.

**Table 1:** Sizes, Polydispersity Index, and Zeta potential of particles synthesized in this study.

Nanoparticle	Size distribution	Mean size	Polydispersity index	Zeta potential
Fe_3_O_4_	17.5 ± 7.5 nm	16 nm	0.3	− 26 ± 4 mv
Fe_3_O_4_@Chitosan	48 ± 27 nm	36 nm	0.3	− 15.5 ± 5.5mv
Fe_3_O_4_@Chitosan@ZIF-8	66.5 ± 33.5 nm	62 nm	0.3	− 12 ± 7mv
Fe_3_O_4_@Chitosan@ZIF-8 @RVG29	180 ± 60 nm	155 nm	0.2	+ 2.5 ± 7.5mv

### Particles' zeta potential

Zeta potential of Fe_3_O_4_, Fe_3_O_4_@Chitosan, Fe_3_O_4_@Chitosan@ZIF-8, and Fe_3_O_4_@Chitosan@ZIF-8@RVG29 varied between – 30 mv to – 22 mv, − 21 mv to − 10 mv, − 19 mv to − 5 mv, and − 5 mv to + 10 mv respectively **(**Table 1**)**. To make sure that the boundaries between the agents remained stable, these values were tested in PBS solution as opposed to water. Consequently, the ions in PBS may lessen the surface charge of the nanoparticles, which would lower the reported zeta potential values. Aside from that, none of the synthesized products appear to have a substantial effect on the zeta potential of the nanoparticles, yielding stable findings over time. Fe_3_O_4_@Chitosan@ZIF-8@RVG29 tended to have positive zeta potential because the peptides had a positive charge. This helps it to cross the blood–brain barrier (BBB)^2,29^ as Barrow et al. demonstrated that a six-fold increase in absorption in mouse mesenchymal stem cells (MSCs) was observed when the charge of 40 kDa coated iron oxide-based nanocomposites was increased from − 1.5 to + 18.2 mV in comparison to neutral coated nanocomposites^30^. Additionally, positively charged nanocomposites can accumulate in tumor areas by counteracting the negative charges on a tumor cell's surface, which strengthens the anticancer effect ^2,29,31^. The here synthesized Fe_3_O_4_@Chitosan@ZIF-8@RVG29 has the almost optimum mean size (150 nm) for neutral and positively charged nanocomposites for BBB bypass^47,48^.

### Particles' magnetic properties

**Figure 3:**
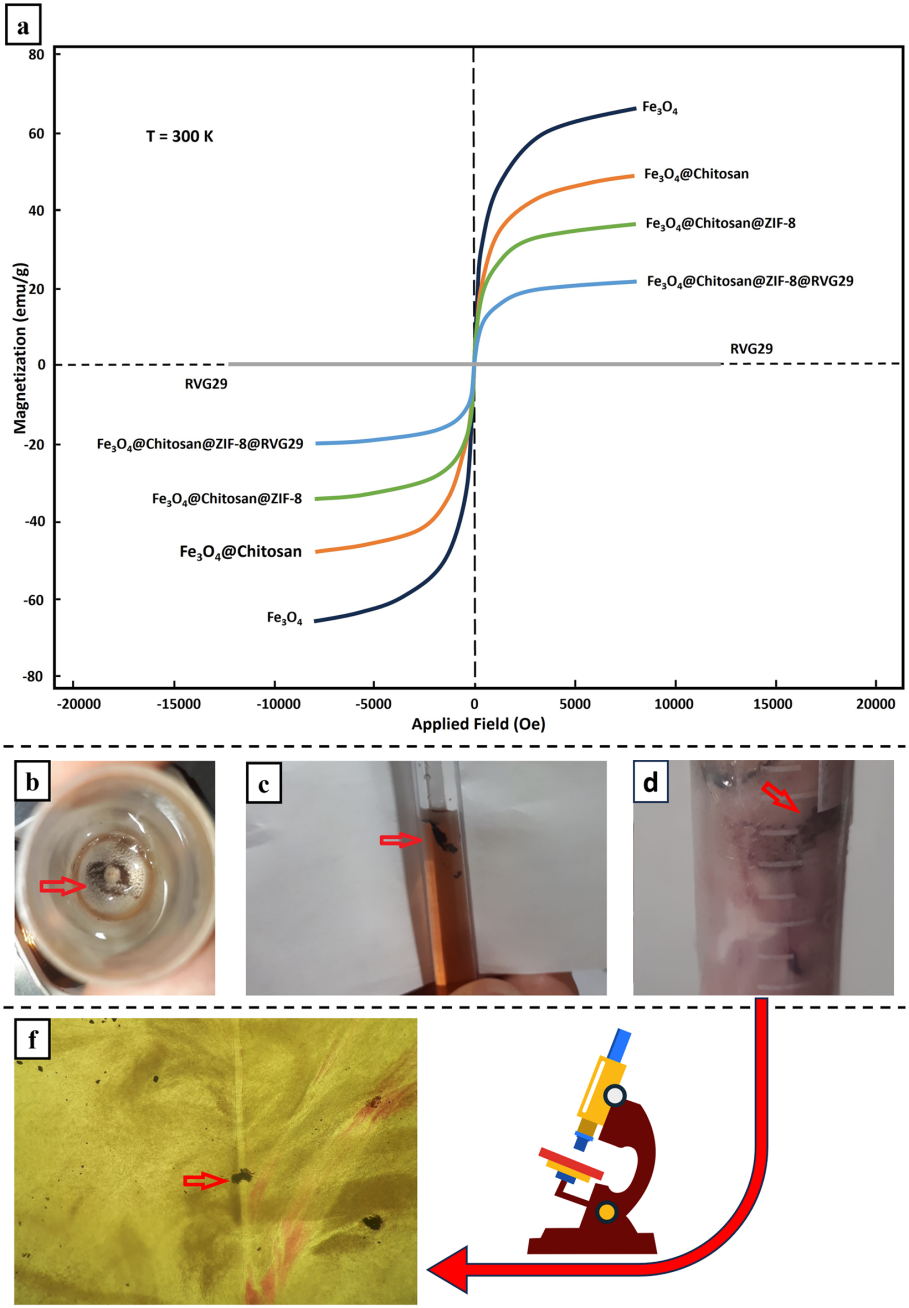
Nanocomposites magnetic behavior. (**a**) VSM pattern of the nanocomposites at 300 K, Movement of Fe_3_O_4_chitosan@Zif-8@RVG29 nanocomosite in (**b**) physiological serum, (**c**) 10% agar, and (**d**) brain f) in the cross-section of brain tissue under Optical Microscope × 100.

The magnetic properties of the products in each nanocomposite synthesizing step were characterized by a Vibrating Sample Magnetometer (VSM). The magnetic curves are shown in Fig. 3a These curves showed that the approximate saturation magnetization value of Fe_3_O_4_@Chitosan, Fe_3_O_4_@Chitosan, and Fe_3_O_4_@Chitosan@ZIF-8@RVG29 nanocomposite is 49.29, 35.96, 21.59 emug^−1^, respectively. The reduction in the saturation magnetization of the nanocomposites compared to that in the pure magnetite nanoparticles (66.3 emug^−1^) is due to coated Chitosan and ZIF-8 nanocrystals and the synergistic effect with RVG29 on its surface. Nevertheless, Fe_3_O_4_@Chitosan@ZIF-8@RVG29 nanocomposite, like the previously synthesized products, exhibited the superparamagnetic characteristic and a high magnetization value, which can be completely, efficiently, and quickly separated from the reaction mixture with an external magnetic field. As shown, the remanent magnetization was equal to zero for all NPs. There was no hysteresis phenomenon and the magnetization and demagnetization curves were coincident.

### Particles' magnetic guidance

The magnetic field guided movement of Fe_3_O_4_@Chitosan@ZIF-8@RVG29 nanocomposites in water (Video S1), physiological serum (Fig. 3b), 10% agar (Fig. 3c), and sheep brain (Fig. 3d) is completely clear. The Optical Microscope image shows the penetration of nanoparticles in the brain tissue (Fig. 3f). All the synthesized magnetic nanoparticles penetration in all mentioned materials under AMF were almost the same with different speeds. Particles with less coating were faster in all materials except for Fe_3_O_4_@chitosan@ZIF-8@RVG29 in the brain tissue; which was faster than the other synthesized particles (drowned faster in tissue). Fe_3_O_4_@chitosan@ZIF-8@RVG29 nanocomposites were poured on the center of the surface of the right hemisphere of the fresh brain tissue and guided into the tissue with an alternating magnetic field. Figure 3f is the section of the center of the treated brain's right hemisphere. Figure 3d resulted from punching another treated brain with an open tube and guiding the nanocomposites to the tube wall with a fixed magnetic field to visualize the depth of penetration made by the final nanocomposites (black dots) guided by AMF. The aim is to magnetically vibrate and control the movement of the reported nanocomposites in the cancerous area of the brain rather than letting the natural brain streams guide them away.

### In vitro cytotoxicity study

**Figure 4:**
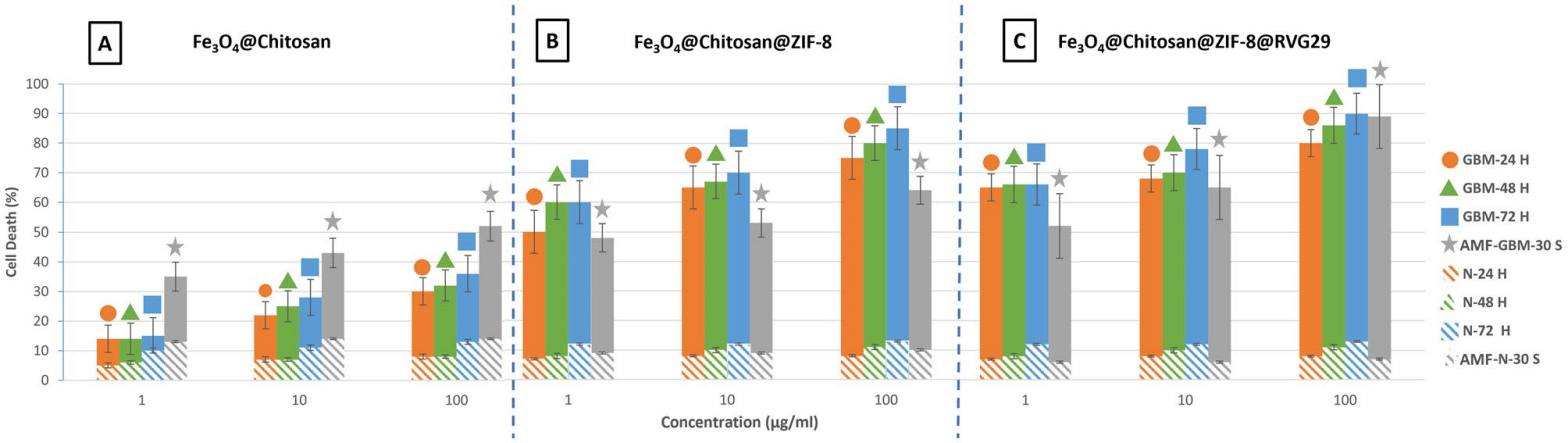
Graph of glioblastoma multiforme) U-87 MG( and normal brain cells death. The death of the cells with Nanocomposites after 24 h was shown by an orange circle, after 48 h by a green triangle, and after 72 h by blow Square. The death of cells after 30 s of exposure to Nanocomposites and AMF treatment was symboled with a gray star. The Hashur area defines Normal brain cell death. Data are presented as mean ± SD (n = 3); p < 0.05.

The in vitro cytotoxicity of the synthesized nanocomposites on U-87 MG cells was evaluated using 3-(4,5-dimethylthiazol-2-yl)-2,5-diphenyltetrazolium bromide (MTT) and enzyme-linked immunoassay assay (ELISA) reader. The 30 s treatment with AMF used in this study had a neglectable effect on normal and U-87 MG cells when used alone. Figure 4 shows the death of glioblastoma multiforme cells (U-87 MG) in comparison with normal brain cells after 24, 48, and 72 h of exposure to Fe_3_O_4_@Chitosan (a), Fe_3_O_4_@Chitosan@ZIF-8 (b), Fe_3_O_4_@Chitosan@ZIF-8@RVG29 (c) without external alternating magnetic field (AMF), and only 3 h of exposure to the mentioned Nanocomposites after 30 s of AMF treatment (in the presents of nanocomposites). Dependence on concentration and time was seen in all three nanocomposites, that is, the higher the concentration and the longer the exposure time, the more cell death was seen. The highest lethality rate of cancer cells (about 90%) was seen in the case of exposure to Fe_3_O_4_@Chitosan@ZIF-8@RVG29 nanocomposite at a concentration of 100 µg/ml after 72 h of incubation at brain temperature (38.5 °C), and the second (89%) in the case of 30 s AMF treatment with the exposure to the same nanocomposite at the same concentration. This is because of their RVG29's glioblastoma multiforme (GBM)-targeting nature, which targets glioma cells by binding to their highly expressed nicotinic acetylcholine receptor (nAchR)^9^ and inducing apoptosis in them^5^. Coating Fe_3_O_4_@Chitosan with ZIF-8 significantly increased its U-87 MG cell line lethality and had a neglectable effect on the brain's normal cell lethality. It is supposed that the released Zn^2+^ during ZIF-8 NPs degradation in GBM cancer cells and the generation of reactive oxygen species in the tumor's acidic microenvironment (TAM) is directly responsible for tumor cell killing^22^. However, 30 s of applying external AMF showed significantly higher GBM cell death in all concentrations of Fe_3_O_4_@Chitosan exposure. This is because of their magnetization superiority in comparison with other nanocomposites Fig. 3a. Only 30 s of applying AMF to GBM cells exposure to 1 µg/ml concentration of Fe_3_O_4_@Chitosan was almost equal to 48 h of their exposure without AMF. However, when exposed to Fe_3_O_4_@Chitosan@ZIF-8 and Fe_3_O_4_@Chitosan@ZIF-8@RVG29, 30 s of applying AMF didn't overcome the AMF-free 24h exposure except for the 100 µg/ml concentration of Fe_3_O_4_@Chitosan@ZIF-8@RVG29 exposure which was almost equal to 72h AMF-free exposure and with taking the standard error bars in account it even overtake it to reach %100 GBM cell lethality. Nanocomposites concentration-related results were seen in all AMF-treated solutions. Moreover, ZIF-8 and RVG29 in the Nanocomposites increased U-87 MG cell line deth in all exposure times and lowered the AMF normal cell killing. The best result in killing GBM cells while preventing normal cell death was obtained by 100 µg/ml Fe_3_O_4_@Chitosan@ZIF-8@RVG29 exposure under 30 s AMF. These results indicate the U-87 MG cell line targeting nature of each coating and RVG29 functionalized nanocomposites superiority in killing U-87 MG cells, and their low cytotoxicity to normal brain cells. Although each coating lowered the Fe_3_O_4_ magnetization (Fig. 3a) and consequently their response to AMF, they still have the superiority in AMF-treated samples by killing more U-87 MG cells and lowering the normal brain cell death.

The mechanism in which the here introduced AMF increases the apoptosis of GBM cells induced by the synthesized nanocomposites and keeping the normal cell safe is supposed to be like the effect of a vibrating stirrer (AMF) in dissolving salt (the nanocomposites) in water (GBM cells) and in oil (normal brain cells). The stirring will make the salt dissolve faster in water but can't dissolve it in oil^49^. Its mechanism is neither magnetic hyperthermia^50^ nor mechanical damage^50^. Unlike magnetic hyperthemia which usually takes 30 or 60 min to kill cancerous cells by increasing the temperature to at least 42 °^51^, this mechanism took only 30 s to destroy more than 89 percent of the GBM cells without a considerable temperature increase. And unlike mechanical damage guided by magnetic field^52^, it keeps the normal cells alive. See the supplementary video S1 online for better visualization. It shows the vibration that the AMF used in this study induces in the Fe_3_O_4_@Chitosan@ZIF-8@RVG29 nanocomposites in water. These vibrations and movements increase the effective collision^53,54^; which results in decreasing the time needed for the nanocomposites to kill the GBM cells. Moreover, the AMF concentrates, vibrates, and keeps the magnetic nanocomposites in the intended tumor site (previously guided to the tumor by a fixed magnetic field) which increases the bioavability^55^ of these nanocomposites.

The nanocomposite introduced in this study optimally combines the properties of used nanomaterials (biocompatible) in the right size to fight GBM^2,29,46–48,56^. Therefore, it brings the highest ratio of lethality of GBM cells to healthy cells. By combining it with the alternating magnetic field introduced in this study, we reduce the treatment time from 72 h to 30 s, and by using a fixed magnetic field, it is possible to direct the nanocomposites to the tumor site and remove them after treatment^2,3^. These Nanocomposites could be monitored with MRI thanks to their Fe_3_O_4_ core^2,24^.

The nanocomposite introduced in this study, in addition to combining and increasing the properties of nanoparticles synthesized in some of the previous studies (mentioned in the introduction), introduces some new properties to fight GBM.

## Methods

### Synthesis of Fe_3_O_4_ nanoparticles

One kilogram of iron ore tailings was combined with 37.5-weight percent hydrochloric acid (HCl) (Sigma-Aldrich Corporation, MO). Pickling was then separated and gathered. To ensure that all of the iron in the filtrate could be in the Fe^3+^ state, the right amount of hydrogen peroxide (H_2_O_2_) (Sigma-Aldrich Corporation, MO) was added. The filtrate was heated to 60 °C and then the proper amount of concentrated ammonia (Sigma-Aldrich Corporation, MO) was added to bring the pH level down to 3.2. Fe consequently precipitated into Fe(OH)_3_ and was separated from tailings. Ultimately, a portion of Fe(OH)_3_ was calcined into Fe_2_O_3_ after being repeatedly cleaned with de-ionized water (Sigma-Aldrich Corporation, MO). The percentage of iron elements was computed and examined, which served as the foundation for the subsequent fabrication of Fe_3_O_4_ nanoparticles. De-ionized water was used to wash the Fe(OH)_3_ precipitate multiple times. Then FeCl_3_ solution was obtained by Fe(OH)_3_ precipitate dissolution with hydrochloric acid.

Fe_3_O_4_ magnetic nanoparticles were synthesized by the chemical co-precipitation method^2^. Fe^3+^ and Fe^2+^ were adjusted to a 1.5:1 molar ratio by adding a measured amount of FeSO_4_·7H_2_O (Sigma-Aldrich Corporation, MO) to the FeCl_3_ solution. The black magnetite was synthesized immediately after adding sodium hydroxide (NaOH) (Sigma-Aldrich Corporation, MO) solution to adjust the pH to 12 under ultrasonic irradiation. The principle of the reaction is:

$$ {\text{Fe}}^{{{2} + }} + {\text{2Fe}}^{{{3} + }} + {\text{8OH}}^{ - } \to {\text{ Fe}}_{{3}} {\text{O}}_{{4}} + {\text{4H}}_{{2}} {\text{O}} $$

The obtained Fe_3_O_4_ precipitate was irradiated in an ultrasonic water bath for 30 min at 65 °C. To purify the synthesized Fe_3_O_4_ particles, they were washed repeatedly with de-ionized water and ethanol (Sigma-Aldrich Corporation, MO) until a pH level of 7 was reached. Then the particles were dried in a vacuum at 74 °C.

### Synthesizing Fe_3_O_4_@CS

Reverse-phase suspension cross-linking method^57^ was used. Fe_3_O_4_ nanoparticles (0.2 g) were distributed in a solution containing 30 ml paraffin and 0.5 ml span− 80 (Sigma-Aldrich Corporation, MO) after being thrice cleaned with 99.5% ethanol. A 15.0 ml solution of CTS in acetic acid with a 2% concentration was then added. For thirty minutes, the suspension was blended using ultrasonic irradiation. The suspension was then added to 3 ml of 25% glutaraldehyde solution and put into a three-neck bottle flask with a mechanical stirrer. After four hours the bottle was placed on a permanent magnet with a surface magnetization of 6000 G, which allowed the chitosan-magnetite nanocomposite particles to be extracted from the reaction mixture. After the magnetic particles settled in 1–2 min, they were cleaned orderly with N, N-dimethylformamide (Sigma-Aldrich Corporation, MO) (three times), ethanol (three times), and deionized water (three times) respectively, and dried in an oven at 50 °C for 12 h.

### Synthesizing Fe_3_O_4_@CS@ZIF-8

First, 0.7 g of nanoparticles that were synthesized in the previous step were added drop by drop to the zinc acetate solution (Sigma-Aldrich Corporation, MO). Then, it was stirred magnetically in 10 ml of methanol for 20 min, after the complete suspension, 0.5 gr of 1,2 dimethyl imidazole (Sigma-Aldrich Corporation, MO) was added drop by drop to the suspension. After stirring for 15 min, the synthesized nanocomposites were washed several times with methanol and PBS (Sigma-Aldrich Corporation, MO). Then, dried in an oven at 100 °C for 12 h and collected for characterization.

### Synthesizing Fe_3_O_4_@CS@ZIF-8@RVG29

One gram of the previously synthesized Fe_3_O_4_@CS@ZIF-8 (limiting reagent) was vortexed with one milliliter of 29 amino-acid peptides generated from the rabies virus glycoprotein (RVG29) (Sequence: YTIWMPENPRPGTPCDIFTNSRGKRASNG) (GenScript, Jiangsu, China) in PBS at 1 mg/ml and incubated for one hour at 37 degrees and collected for characterization.

### FE-SEM and TEM

FE-SEM was used to analyze the topographic details on the surface of the nanoparticles. images were obtained with an accelerating voltage of 10 kV using a Tescan model MIRA3 (Tescan company, Czech Republic). All samples were coated with 8nm gold using a gold sputter Leica EM SCD005 instrument before imaging. The morphology of synthesized Fe_3_O_4_ was analyzed by using a transmission electron microscope (TEM, Zeiss EM10C, Oberkochen, Germany) at 100 kV.

### DLS

Using a particle size analyzer (HORBIA SZ100 Z, Japan), dynamic light scattering (DLS) was used to determine the mean hydrodynamic diameter of the nanoparticles. In PBS, each sample was diluted by a factor of 1:400. Every measurement was taken at 25 °C and a 90° angle of light incidence. The polydispersity index was calculated based on the width of the particle size distribution, and the hydrodynamic diameter carried by a Gaussian distribution.

### Zeta potential analyzer

Using a zeta potential analyzer (HORIBIA SZ100 Z, Japan), the electrophoretic mobility of the nanoparticles was measured to determine their zeta potential. Every sample was diluted by a factor of 1:400 in PBS, and all measurements were done at 25 °C.

### FTIR

FTIR was done to verify the presence of absorption bands in samples, as well as the presence of each material added to the surface of the nanocomposite. Previously, the materials were lyophilized in a LyoQuest − 85 freeze dryer (Telstar, Terrassa, Spain) at − 85°C and 0.76 Torr. The lyophilized nanoparticles' infrared spectra were gathered using an FTIR Spectrometer model 8400 from SHIMADZU (Ottawa, Canada).

### MTT assay

A cell suspension from U-87 MG (a Glioblastoma multiform cell line) in RPMI1640 culture medium enriched with 10% FBS and 1% antibiotics was provided from Pasteur Institute, Tehran, Iran. 100 µl of Fe_3_O_4_@Chitosan, Fe_3_O_4_@Chitosan@ZIF-8, and Fe_3_O_4_@Chitosan@ZIF-8@RVG29 in the concentration of 1, 10, and 100 µg/ml with two repetitions for the periods of 24, 48, and 72 h were poured in the wells of 96 wells with a sampler under a sterilized hood and 100 µl of the cell suspension (2.5 × 10^4^ cells/well) were immediately poured into all the microplate wells. After 24 h, 10 µl of MTT solution was poured into the first 18 wells on the left side and their bottom row, and after three hours, MTT metabolic product—formazan was dissolved in 100 µl dimethyl sulfoxide (DMSO) and the light absorption was read at 490 and 630 nm with an ELISA reader. After 48 and 72 h, the same was done for the second and third 18 wells, and the results were recorded.

I repeated the same procedure for the normal brain cells (Pasteur Institute, Tehran, Iran). An additional MTT assay under 30 s of alternating magnetic field (AMF) and without AMF (control) for normal and U-87 MG brain cells with each nanocomposite in microtubes without further incubation (only 3 h of incubation after adding the MTT solution for the ELISA reader) was done. The control groups (cells treated for 30 s + 3 h with only nanocomposites) showed neglectable death.

### VSM

The magnetic behavior of 0.2 gr of each synthesized nanocomposite was characterized as a function of 0 to a 1-T magnetic field and 300-K temperature based on Faraday's Law of Induction^2,58^ using MDKF-FORC/VSM MEGNATIS-DAGHIGH KASHAN CO.

### Treating with fixed and alternating magnetic fields

This test was performed on water, physiological serum, 10% gel, and sheep brain. For this purpose, 10 ml of 100 µg/ml of synthesized nanocomposite in PBS was added to the above-mentioned materials in tubes and treated with a Fixed magnetic field (Y 28, Jai-Mag, India) and an alternating magnetic field (AMF) (2400 revolutions per minute, 5.3 mN/m Torque, 50–60 Hz Frequency) (Atlas, Iran). By moving the tubes back and forth, the movement of nanocomposites in the magnetic field was photographed and evaluated. For a more detailed investigation, Sections from the brain tissue used were prepared and the possible penetration of nanocomposites and their accumulation in the tissue and cells were evaluated with an optical microscope.

### Statistical analysis

Statistical analysis was performed using SPSS software version 27.0.1. The measurements were repeated three times and data were expressed as mean ± SD. Data were analyzed using one-way analysis of variance (one-way ANOVA) followed by Tukey’s test. A *p*-value lower than 0.05 was considered statistically significant.

### Supplementary Information

Supplementary Video S1.

## Data Availability

The data presented in this study are available on request from the corresponding author. The data are not publicly available as they are part of ongoing research.
